# Correction: Abou-ElNaga et al. Novel Nano-Therapeutic Approach Actively Targets Human Ovarian Cancer Stem Cells after Xenograft into Nude Mice. *Int. J. Mol. Sci*. 2017, *18*, 813

**DOI:** 10.3390/ijms26010408

**Published:** 2025-01-06

**Authors:** Amoura Abou-ElNaga, Ghada Mutawa, Ibrahim M. El-Sherbiny, Hassan Abd-ElGhaffar, Ahmed A. Allam, Jamaan Ajarem, Shaker A. Mousa

**Affiliations:** 1 Zoology Department, Faculty of Sciences, Mansoura University, Mansoura 35516, Egypt; amoura55555@gmail.com (A.A.-E.); ghada.adel.mutawa@gmail.com (G.M.); 2 Center for Materials Science, Zewail City of Science and Technology, Cairo 12588, Egypt; ielsherbiny@zewailcity.edu.eg; 3 Clinical Pathology Department, Faculty of Medicine, Mansoura University, Mansoura 35516, Egypt; haabdelghaffar@yahoo.com; 4 Zoology Department, College of Science, King Saud University, Riyadh 11451, Saudi Arabia; aallam@ksu.edu.sa (A.A.A.) ; jajarem@ksu.edu.sa (J.A.); 5 Zoology Department, Faculty of Science, Beni-Suef University, Beni-Suef 62511, Egypt; 6 The Pharmaceutical Research Institute, Albany College of Pharmacy and Health Sciences, Rensselaer, NY 12144, USA

In the original publication [1], there were mistakes in Figures 4–6. Figure 4, which presented the histopathological examination results, erroneously included sections from all groups, including the control group, which was deemed unnecessary for the intended analysis. Figure 5, specifically panel 5b(III), depicted the immunohistochemistry results for Caspase-3 and P53, but did not accurately represent the intended data due to image artifacts. The published Figure 6 showed both the PCR analysis (Figure 6a) and RT-q.PCR analysis (Figure 6b) of the investigated genes.

The corrected Figure 4, Figure 5 and Figure 6 appear below. The revised Figure 4 includes only the sections from the vehicle and treated groups (plain NPs, free PTX, and PTX-loaded FA/PLGA NPs), providing a more focused and relevant representation of the histopathological findings associated with the different treatment modalities. The updated panel 5b III in Figure 5 reflects the intended findings more accurately. The updated Figure 6 shows only the RT-qPCR analysis, which is sufficient to demonstrate the quantitative expression of the genes without duplicating the results.

The following correction has been made to Section 2. Results, 2.3.2. Histopathological Examination of Tumor and Main Organ Sections:

Sections of the adjacent areas of the excised tumors after formalin fixation, paraffin embedding and histopathological examination are shown in Figure 4a. Aggregates of spindle shaped cells clearly appeared (arrow) in sections of the tumors injected with plain NPs. We noted the disappearance of such spindle-shaped cells in sections of the tumors treated with free PTX. On the other hand, reactive lymphatic follicles appeared (arrow) in sections of the tiny nodules that remained in the skin at the site of the injection of PTX-loaded NPs.

Sections of the main organs showed no clear pathological variation between the groups except for the intestine (Figure 4b). We noted a clear rupture of the intestine (arrow) in the case of free PTX treated mice, whereas the intestine sections of PTX-loaded NPs-treated mice were completely free of any damage.

The following correction has been made to Section 2. Results, 2.3.4. mRNA Expression of Apoptotic, Chemo-Resistant and Tumor Suppressor Genes:

RT-qPCR analysis was applied to detect the expression of apoptosis-related cysteine peptidase-9 (*caspase9*), apoptosis-related cysteine peptidase-3 (*caspase3*), Tumor suppressor 53 (*TP53*), ATP-binding cassette sub-family G-2 (*ABCG2*) and Multidrug resistance-1 (*MDR1*) in the tumor sections treated with PTX-loaded NPs in comparison with free PTX (Figure 6). Results showed no clear variation in the expressions of apoptotic genes (*Caspase-9* and *Caspase-3*) and tumor suppressor gene (*TP53*) between tumors treated with PTX-loaded NPs and free PTX; however, PTX-loaded NPs expressed these genes 0.8-fold, 2-fold and 4-fold, respectively, compared to the free PTX. Additionally, PTX-loaded NPs expressed chemoresistant genes (*ABCG2* and *MDR1*) 256-fold and 512-fold greater, respectively, than free PTX.

The authors state that the scientific conclusions are unaffected. These corrections were approved by the Academic Editor. The original publication has also been updated.

## Figures and Tables

**Figure 4:**
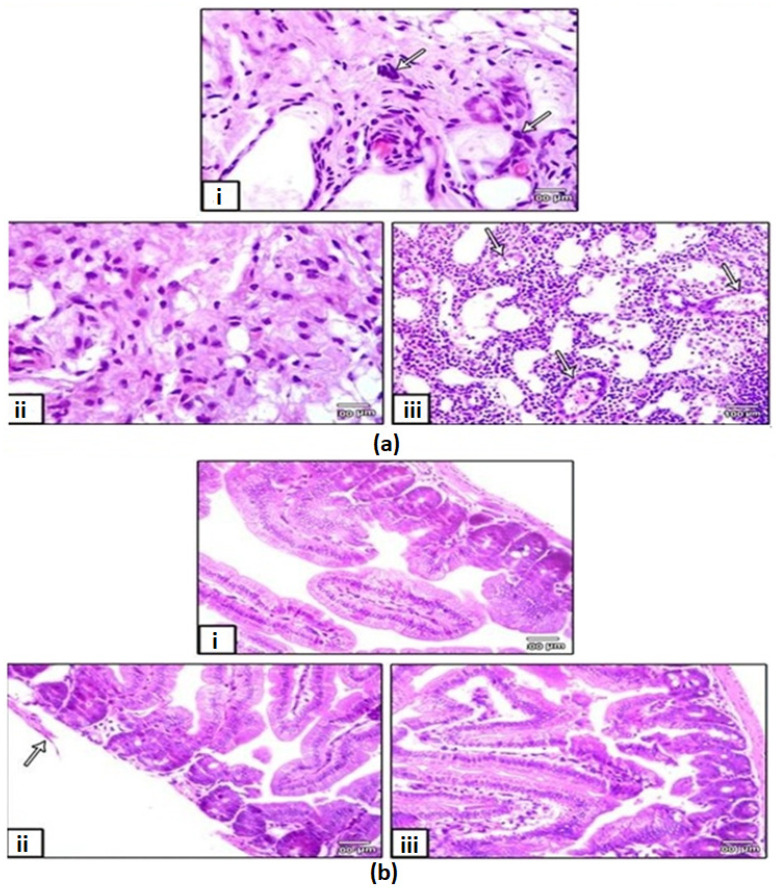
Histopathological examination. (**a**) Tumor and (**b**) intestine sections after the treatments (H&E staining): (**i**) plain NPs; (**ii**) free PTX; (**iii**) PTX-loaded FA/PLGA NPs. In (**a**), arrows in (**i**) point to aggregates of cancer cells and those in (**iii**) to reactive lymphoid follicles; In (**b**), the arrow in (**ii**) points to a clear rupture in intestinal serosa.

**Figure 5:**
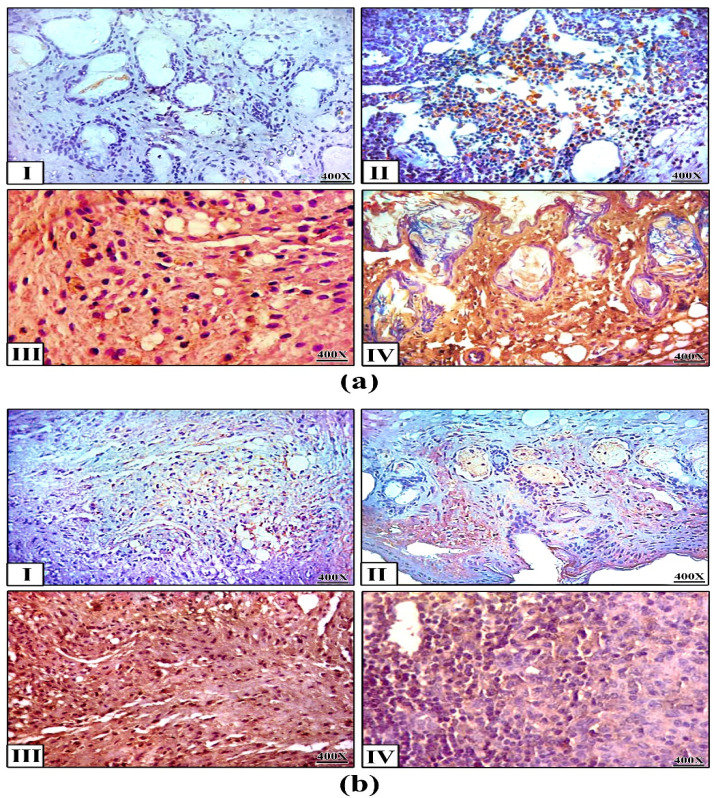
Immunohistochemistry. Expression of (**a**) Caspase-3 and (**b**) P53 in tumor tissues after the treatments (400×). **(I)** saline, (**II**) plain NPs, (**III**) free PTX, (**IV**) PTX-loaded FA/PLGA NPs.

**Figure 6:**
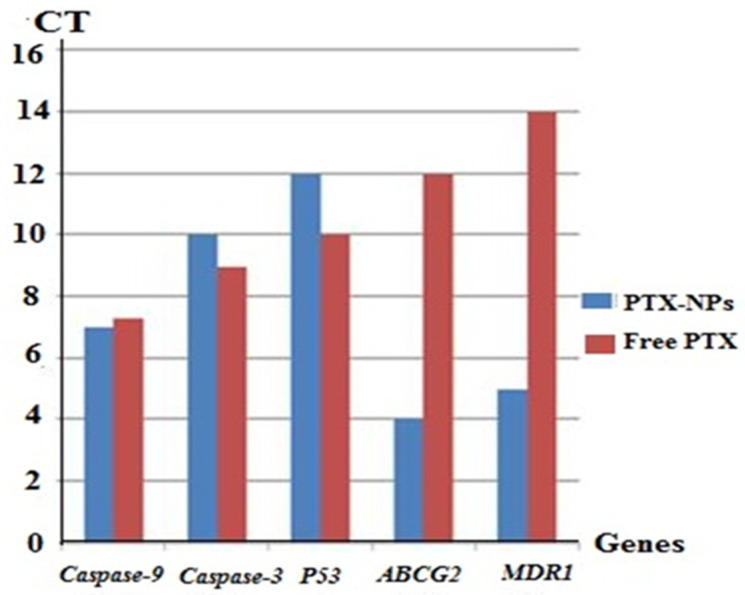
RT-q.PCR analysis of Caspase 9, Caspase3, TP53, ABCG2 and MDR1 m.RNA. Lane 1: PTX-loaded FA/PLGA NPs treated tumors, Lane 2: free PTX. *GAPDH* was used as an internal control.
